# Role of Insulin in the Type 2 Diabetes Therapy: Past, Present and Future

**DOI:** 10.5812/ijem.7551

**Published:** 2013-07-01

**Authors:** Carlo Maria Rotella, Laura Pala, Edoardo Mannucci

**Affiliations:** 1 Obesity Agency, University of Florence Medical School, Careggi Teaching Hospital, Firenze, Italy; 2 Endocrinolgy Unit , University of Florence Medical School, Careggi Teaching Hospital, Firenze, Italy; 3 Diabetes Agency, University of Florence Medical School, Careggi Teaching Hospital, Firenze, Italy

**Keywords:** Insulin Therapy, Type 2 Diabetes, Cancer, Cardiovascular Effects

## Abstract

**Context:**

Since 2006 a relevant number of therapeutical algorithms for the management of type 2 diabetes have been proposed, generating a lively debate in the scientific community, particularly on the ideal timing for introduction of insulin therapy and on which drug should be preferred as add-on therapy in patients failing to metformin. At the moment, there is no real consensus. The aim of the present review is to summarize established knowledge and areas for debate with respect to insulin therapy in type 2 diabetes.

**Evidence Acquisition:**

In type 2 diabetic patients, insulin represents a therapy with a long and well-established history, but, considering the modern insulin therapy, several points must be carefully examined. The role played by the introduction of insulin analogues, the choice of insulin regimens, the ongoing debate on insulin and cancer, the cardiovascular effects of insulin, the role of insulin on β-cell protection and the actual clinical perspective in the treatment of the disease. Nevertheless, still many exciting expectations exist: the new insulin analogues, the technological options, the inhaled and oral insulin and the issue of transplantation.

**Conclusions:**

Although insulin is the more potent hypoglicemic agent, the availability of a wider spectrum of therapeutic agents, many of which are better tolerated than insulin, has reduced the field of application for insulin treatment; presently, insulin is used only in those who cannot maintain an adequate glycemic control with other drugs. Furthermore, a lively research activity is currently ongoing, in order to make insulin therapy even safer and simpler for patients.

## 1. Context

Since 2006 a relevant number of therapeutic algorithms for the management of Type 2 Diabetes (T2DM) have been proposed, generating a lively debate in the scientific community, particularly on the ideal timing for introduction of insulin therapy and on which drug should be preferred as add-on therapy in patients failing to metformin. Actually, there is no real consensus. The aim of the present review is to summarize established knowledge for debate with respect to insulin therapy in T2DM.

### 1.1. The Past: A Therapy with a Long and Well-Established History

The availability in 1923 of the first insulin preparations for use in humans completely changed the natural history of Type 1 diabetes, enabling physicians to save the life of those patients. In those days, the use of insulin in patients with T2DM was reserved to those individuals who were not able to follow a diet and had severe hyperglycemia. The compliance of patients to insulin, initially represented only by short-acting regular porcine or bovine insulin, was generally poor, and this resulted in important excursion of glycaemia. Only in 1950 a commercial insulin preparation with longer duration of action, the Neutral Protamine Hagedorn (NPH) insulin, became available. NPH insulin became popular for the therapy of T2DM, because they allowed an acceptable glycemic control with only one or two daily injections. Tolbutamide, the first oral antidiabetic drug, appeared only in 1957. Thus, for 34 years the only available glucose-lowering drug was insulin, irrespective of diabetes type.

After the introduction of the first sulfonylurea, many other secretagogues were synthesized such as biguanides. For many years, the typical therapy for T2DM was mainly based on sulfonylureas, with a biguanide (mostly fenformin) added in the case of insufficient control; in fact, sulfonylureas alone did not allow to maintain satisfactory glycemic levels in the majority of patients after a few years from the initiation of therapy (1, 2). In case of failure to two oral drugs, insulin therapy was added, often starting as a single shot of bedtime NPH bedtime, and finally oral drugs were discontinued and the patient was treated with insulin only.

In the 1980s, the concept of good metabolic control was very flexible and most of the patients were considered as target with HbA1c levels between 8 and 9 %. A real revolution in the therapy of T2DM occurred after the publication of data of the UKPDS (3, 4), showing that the intensification of blood glucose control with a sulfonylurea or insulin, significantly reduced the risk of long-term diabetic complications. Based on the results, the idea that the attainment and maintenance of near- euglycemia could be beneficial in patients with T2DM gained ground in the scientific community. Some studies also suggested that early intensive insulin therapy in patients with newly diagnosed T2DM could have outcomes for recovery and maintenance of β-cell function and protracted glycemic remission, if compared with treatment with oral hypoglycemic agents (5). The drive toward more ambitious therapeutic targets (6), which often required the addition of insulin to oral drugs, was limited by the occurrence of hypoglycemia. In the 1990s, rapid and long-acting insulin analogues became available, facilitating the attainment of more ambitious goals. The improved safety of insulin therapy, due to the introduction of analogues, together with the expectations of β-cell protection, led to the recommendation of early insulin supply in the natural history of T2DM, which was supported by many diabetologists in recent years.

### 1.2. The Present: Modern Insulin Therapy for T2DM

The introduction of short-acting insulin analogues has remarkably improved post-prandial glucose control, both in type 1 (7) and T2DM (8, 9). Furthermore, the use of rapid acting insulin analogues has allowed a greater flexibility in timing and size of meals, avoiding the need for undesired snacks and thus improving patients’ quality of life (10).

NPH insulin was the most widely used retarded insulin prior to the introduction of long-acting analogues. NPH for its duration of action requires two daily administrations in order to provide an adequate basal supply. The first available long-acting analogue, glargine, represented a substantial improvement: it had a duration of action compatible with a once daily administration in the majority of patients and a greater reproducibility with lower hypoglycemic risk. Randomized trials demonstrated that glargine, versus NPH, produced a similar degree of glycemic control with lower risk of nocturnal hypoglycemia both in type 1 (11) and T2DM (12). Another long-acting analogue, detemir, has a similar profile of glargine, with best effect on weight gain (12, 13), but it requires a twice-daily administration in the majority of patients (13).

Analogues, although more expensive, have progressively become the therapy of choice in most countries. The adoption of analogue-based treatment schemes for basal-bolus therapy has allowed for greater flexibility in diet and lifestyle. Furthermore, the lower risk of nocturnal hypoglycemia with glargine and detemir has facilitated an earlier use of basal insulin in T2DM. 

## 2. Evidence Acquisition

### 2.1. The Choice of Insulin Regimens

Type 1 diabetes is usually treated with mealtime boluses of rapid-acting analogues and one or two daily injections of long-acting analogues. Such a scheme can be used also in T2DM often in combination with oral drugs. Many authors suggest that the addition of basal insulin is the preferable approach in patients failing to oral therapies (14, 15). Although a single bedtime administration of a long-acting analogue is convenient, the only available large-scale trial comparing different insulin regimens in T2DM failed to show any major difference across treatment groups (16). T2DM is a very heterogeneous condition; we should expect that patients with predominantly fasting hyperglycemia would have a better response to basal insulin only, while those with mainly post-prandial hyperglycemia are obvious candidates for prandial insulin. As a consequence, basal only, bolus only, or basal-bolus can be chosen on the basis of patients’ daily glucose profiles, tailoring insulin therapy on individual needs. 

### 2.2. Insulin and Cancer: An Ongoing Debate

Insulin is a growth factor, which stimulates the proliferation of normal and malignant cells. It is mitogenic, but not mutagenic; furthermore, a growth-stimulating factor can promote the proliferation of pre-existent transformed cells, converting a subclinical in situ malignancy in a clinically relevant cancer.

**Figure 1.**
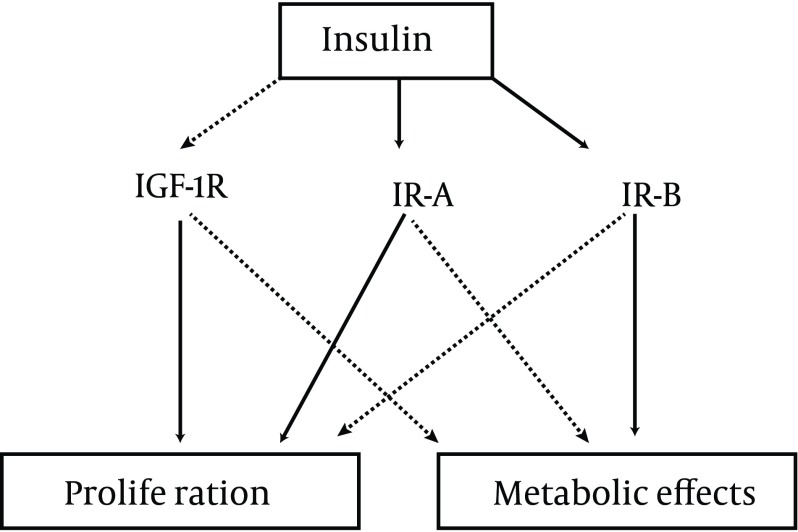
Effects of Insulin on Different Receptors IGF-1R: insulin-like growth factor-1 receptor; IR-A: type A insulin receptor; IR-B: type B insulin receptor.

Insulin is capable of stimulating cell growth via the interaction with multiple receptors (Figure 1).

Insulin is a weak agonist for the Insulin-Like Growth Factor 1 (IGF-1) receptor, the stimulation of which has a powerful effect on cell proliferation. When the IGF-1 receptor is overexpressed and in conditions of hyperinsulinemia, the effect of insulin on the IGF-1 receptor could become more relevant (17). Insulin stimulates cell growth also through the stimulation of the IR-A insulin receptor subtype which has a greater effect on proliferation than the IR-B receptor (18). However, even the stimulation of IR-B insulin receptors enhances cell proliferation (19). This means that the glucose-lowering effects of insulin cannot be entirely separated by its growth-promoting actions.

The effects of insulin on cell growth in vitro are typically dose-dependent. From a clinical perspective, it is important to establish whether insulin concentrations are sufficient to induce a higher risk of cancer. Epidemiologic studies show that, among patients with T2DM, those receiving insulin therapy have a higher incidence of overall malignancies (20). The estimates of risk, which are dependent on insulin doses and duration of insulin treatment (21), vary across studies (20). All those epidemiological studies are affected by prescription bias: patients receiving a prescription for insulin therapy can be affected by conditions which increase the risk of cancer and cannot be accounted for in statistical adjustments. Consequently, it would be preferable to refer to randomized trials, but so far, there is only one large scale trial with appropriate size and duration which compared insulin with other therapies, reporting data on the incidence of malignancies: the ORIGIN trial which failed to detect any difference between treatment groups after 6 years of follow-up (22). It was performed on recent-onset diabetes, using relatively low doses of insulin (less than 0.35 UI x kg/day on average) and it did not have a sufficient statistical power to detect between-group differences in the incidence of individual types of cancer. Epidemiological studies probably overestimate the impact of insulin therapy on the incidence of cancer, whereas experimental studies suggest that high doses of insulin could facilitate the onset of at least some forms of malignancies. However, concerns on cancer should not prevent physicians from prescribing insulin, even at high doses, when an adequate glycemic control cannot be reached otherwise: the benefit of metabolic control certainly exceeds the potential risks for malignancies.

The possibility that insulin analogues have a different effect on the incidence of cancer than human insulin, has been a reason for concern since the 1990s, when the clinical development of the rapid-acting analogue AspB10 had to be terminated because of the risk for malignancies - an effect attributed to a lower dissociation rate from the insulin receptor and/or to a higher affinity for the IGF-1 receptor (23). Glargine also has a greater affinity for the IGF-1 receptor than human insulin, producing a greater proliferative effect in vitro (24). However, this potential risk is mitigated by the metabolic clearance of glargine, which is converted into active metabolites with a low affinity for the IGF-1 receptor. Despite some alarming results from epidemiological studies (25, 26), other surveys have excluded any major effect of glargine on overall cancer rate (27-29). However, based on the results of observational studies, longer-term treatment with higher doses of glargine could be associated with a greater risk of some malignancies in comparison with NPH (30-32). The interpretation of epidemiological studies requires caution, but the results of ORIGIN are not conclusive (22).

### 2.3. Cardiovascular Effects of Insulin: Beneficial or Detrimental?

**Figure 2.**
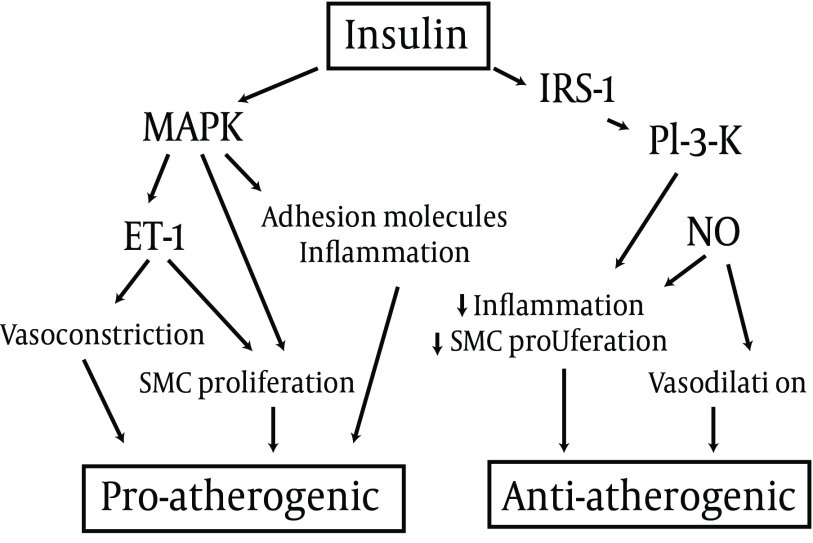
Cardiovascular Effects of Insulin: Molecular Mechanisms MAPK: MAP kinase; IRS-1: insulin receptor substrate 1; PI-3-K: phosphatidy l inositole 3 kinase; ET-1: enothelin-1; NO: nitric oxide; SMC: smooth muscle cell.

The debate on the cardiovascular effects of insulin has been ongoing for many years. Based on experimental studies, insulin appears to have both pro-atherogenic and anti-atherogenic effects (Figure 2).

In healthy individuals, insulin has vasodilator and vasoprotective actions, but in insulin resistant subjects, the opposite effects could prevail (33). In epidemiological studies, patients with T2DM receiving insulin treatment show a higher incidence of cardiovascular disease; furthermore, in the ACCORD trial, intensified therapy was associated with increased cardiovascular mortality (34), prompting some authors to recommend caution in the prescription of insulin (35). However, in observational studies, insulin-treated patients have a greater severity of disease and a higher comorbidity, which cannot be entirely eliminated by statistical adjustments and which may account for poorer outcomes. On the other hand, the increased mortality in the ACCORD study could be the consequence of an elevated incidence of severe hypoglycemia, rather than a negative effect of insulin per sè (8). Conversely, a randomized trial in patients with myocardial infarction showed a remarkable reduction of cardiovascular morbidity and mortality associated with intensified insulin therapy (36), inducing many specialists to postulate insulin as the treatment of choice in patients with previous cardiovascular events. However, in the cited trial, the improvement in glycemic control could have been responsible for the observed beneficial effect on cardiovascular risk.

Trials that followed failed to show any specific cardioprotective effect of insulin. In the DIGAMI-2 trial, intensive insulin treatment did not show any advantage over conventional therapy in patients with T2DM and myocardial infarction (37). Similarly, in the BARI-2D trial, in patients with ischemic heart disease, insulin provision was not superior to insulin sensitizers in the prevention of new events (38). More recently, the ORIGIN trial showed that, in patients with recent-onset T2DM, basal insulin does not reduce cardiovascular morbidity and mortality in comparison with oral drugs (22). Taken together, these data suggests that the cardioprotective effect of insulin observed in earlier trials (36) is determined by the improvement of glycemic control rather than by a specific action of insulin per sè. This is confirmed by the lack of any effect of glucose-insulin infusions in the acute phase of myocardial infarction in patients without diabetes (39). On the other hand, the ORIGIN trial demonstrates the cardiovascular safety of insulin therapy, provided that the incidence of hypoglycemia remains reasonable. 

### 2.4. Is Insulin β-cell Protective?

T2DM is characterized by a progressive loss of β-cell mass and function, leading to a deterioration of glycemic control. It has been hypothesized that insulin therapy in earlier stages of the natural history of diabetes could protect β-cells, preserving insulin secretory capacity and glycemic control over the time, as suggested by some small trials (5). In a sample of patients with impaired glucose tolerance and/or impaired fasting glucose enrolled in the ORIGIN trial, glargine reduced the incidence of diabetes by about 30% (22); this result was similar to that observed with metformin (40) and acarbose (41), and smaller than that reported for thiazolidinediones (42) or lifestyle interventions (40, 43). Furthermore, the protection conferred by insulin treatment seemed to be already fading after a few months (22). Based on these results, insulin per sè does not seem to have a relevant, glucose-independent β-cell protective effect. Therefore, there is no reason to anticipate insulin therapy in an attempt at modifying the natural history of T2DM.

## 3. Results

**3.1. Insulin Therapy Today: the Clinical Perspective**

Insulin remains the most effective glucose lowering therapy in T2DM. For this reason, it is recommended in cases of severe hyperglycemia, particularly when ketonuria or weight loss is reported (14, 15). In all other cases, other alternative therapies might be more convenient (Table 1).

**Table 1.** Benefits and Harms of Currently Available Drugs for Type 2 Diabetes

	Insulin	Metformin	SU/Glinides	AGI	TZD (Pioglitazone)	DPP-4 Inhibitors	GLP-1 Agonists
**Short-term efficacy on glucose**	+++	++	++	+	+/++	+	++
**Long-term efficacy on glucose**	+++	++	+	+	++	?	?
**Risk of hypoglycemia**	+++	-	++	-	-	-	-
**Effect on body weight**	↑ ↑	-/ ↓	↑	-/ ↓	↑ ↑	-	↓ ↓
**Effect on cardiovascular risk**	-	-/ ↓	↑ (?)	-/ ↓	↓	↓ (?)	↓ (?)
**Gastrointestinal side effects**	-	++	-	++	-	-	++
**Other adverse events**	+a	-	-	-	++b	-	+c
**Need for regular SMBG**	+++	-	+	-	-	-	-
**Cost (including that for SMBG)**	+++	+	++	+	++	++	+++
**Impact on quality of life**	+++	+	+	++	++	+	++

^a^Potential risk of cancer

^b^heart failure, bone fractures, bladder cancer

^c^potential risk of pancreatitis

Abbbreviaions: SU, sufonlylureas; AGI, alpha glucosidase inhibitors; TZD, thiazolidinediones; DPP-4, dipeptidyl peptidase 4; GLP-1, glucagon-like peptide-1; SMBG, self-monitoring of blood glucose

Current guidelines recommend metformin as first-line therapy for T2DM. Considering its good short- and long-term glucose-lowering efficacy, fair tolerability, remarkable safety, and low cost, metformin is the most suitable option for patients with T2DM, unless contraindicated (14). When metformin alone is insufficient to reach a satisfactory control, another drug should be added. Although insulin is a possible option, other drugs seem to be preferable. In fact, pioglitazone, DPP-4 inhibitors, GLP-1 agonists and acarbose, unlike insulin (22), could all have some beneficial effect on cardiovascular risk (44-47); furthermore, they do not induce hypoglycemia, which is negative cardiovascular mortality (48) and they require no regular blood glucose self-monitoring. The most suitable place for insulin in the treatment algorithm for T2DM is the failure to two or three non-insulin drugs.

When an insulin therapy is initiated in a patient with T2DM, an attempt should be made at minimizing the adverse effects of treatment and most notably hypoglycemia and weight gain. The use of analogues in the place of human insulin and the choice of a scheme tailored on glucose profiles of individual patients are effective tools for reducing hypoglycemic risk. The combination of insulin with metformin limits weight gain, reduces insulin doses (15), and tampers potential risks of cancer associated with high-dose insulin (49). DPP-4 inhibitors and GLP-1 receptor agonists could also be conveniently combined with insulin, to reduce insulin doses and limit weight gain, although available evidence is limited (50). On the other hand, the use of sulfonylureas in patients treated with insulin is questionable, as it increases the risk of hypoglycemia (51), whereas thiazolidinediones are associated with a higher risk of heart failure when combined with insulin (52).

### 3.2. The Future: Still Many Exciting Expectations

#### 3.2.1. New Insulin Analogues

The duration of action of available long-acting insulin analogues (glargine and detemir) is not sufficient to warrant an adequate supply of basal insulin over 24 hours with one daily injection in all patients. Furthermore, despite improved reproducibility, some fluctuation in insulin levels across different days in the same individual persist, leading to some hypoglycemic episodes. Degludec is a novel insulin analogue, capable of forming multi-hexamer chains of insulin in subcutaneous depots, leading to a longer duration of action (terminal half-life > 25 h and activity > 40 h) (53). Degludec, which has an elevated dissociation rate from the insulin receptor, a low affinity for IGF-1 receptors, has a low mitogenic activity in vitro (54). In comparison with glargine, degludec is characterized by a lower of overall and nocturnal hypoglycemia, despite a similar efficacy on glucose control (55). Another approach to the prolongation of action is the conjugation of insulin with polyethylene glycol (PEG). A PEGylated lispro insulin has been shown to have a similar efficacy as that of glargine, with longer duration of action, lower hypoglycemic risk, and smaller weight gain (56), which should be confirmed in larger studies.

Another area of research is that of ultra-fast acting insulin analogues, aimed at a more accurate reproduction of physiologic prandial insulin release. Several such formulations have been developed (57, 58), but their clinical superiority over currently available rapid-acting insulin analogues in type 2 diabetes is questionable, and it has never been demonstrated so far.

#### 3.2.2. Technological Options

In patients on basal-bolus insulin therapy, an alternative to multiple daily injections is continuous subcutaneous insulin infusion (CSII) using insulin pumps. This option, which is a useful tool in type 1 diabetes (59), is still questioned in T2DM (60, 61). In fact, there is no evidence to date that CSII improves glycemic control in patients with type 2 diabetes in comparison with multiple daily injections (62). Technological advancements in insulin such as “patch-pumps” which do not need an external catheter (63), could improve patients’ acceptance and therefore clinical outcomes also in type 2 diabetes. A further improvement may be represented by the development of an integrated continuous glucose monitoring (CGM) and pump system. A fully functional closed-loop system includes three essential parts: a pump for insulin delivery, a CGM system or a sensor that can keep continuous track of blood glucose and an algorithm that determines the insulin delivery amounts and rates (64). Pilot short-term studies on the efficacy of such closed loop systems are encouraging, at least for type 1 diabetes (65). Further research is needed to verify whether this approach is also potentially useful in T2DM. 

### 3.3. Inhaled and Oral Insulin

The idea of providing insulin through routes different from traditional subcutaneous injection has always been considered very appealing. In fact, patients’ refusal of injectable is one of the current obstacles to insulin therapy. Inhaled micronized insulin, after having been registered in the US, was withdrawn because of side effects and adverse events – most notably respiratory insufficiency and lung fibrosis (66). Despite this failure, other similar systems, including oral insulin, are currently under development (67), producing formulations with a shorter action than subcutaneous regular insulin (68). Although preliminary studies in type 2 diabetes (69, 70) and impaired glucose tolerance (71) were promising, a much greater amount of data should be produced in order to be confident in the long-term safety of this approach. 

### 3.4. In Search of the Final Solution: Transplantation and Beyond

Pancreas transplantation is known to produce a complete remission of type 1 diabetes in the majority of cases, provided that an appropriate immunosuppressive therapy is applied. Very few studies are available for pancreas transplantation in T2DM, in conjunction with kidney transplantation, with inconclusive results. In fact, isolated kidney transplantation from living donor in patients with type 2 diabetes seems to warrant better outcome than simultaneous pancreas-kidney transplantation from deceased donor; however, the addition of pancreas improves the outcome of kidney transplantation from deceased donor (72). Based on these results, in T2DM, pancreas transplantation is an option, in combination with kidney, only in patients with renal failure for whom a living kidney donor is unavailable.

A more promising approach is represented by gene therapy, i.e. the introduction of functioning therapeutic genes in some of the patients’ cells. This procedure is based on the use of viral vectors in which the virus’ capside functions as carrier for the desired gene, together with appropriate promoters. Several potential targets for gene therapy can be hypothesized for T2DM; for example, gene therapy with the exenatide gene in rodents has therapeutic effects not dissimilar from those of exogenous exenatide administration (73). One of the most obvious targets for gene therapy is insulin: using this approach, it is possible to induce insulin synthesis and secretion in different organs, obtaining a therapeutic result. Insulin gene therapy has been shown to induce remission of hyperglycemia within a few days of treatment in rodent models of type 1 diabetes (74). However, such a therapy has never been tested in models of T2DM, or in animals more similar to humans. 

## 4. Conclusions

Historically, insulin has been the first available therapy for diabetes. Despite the introduction of many alternative glucose-lowering treatments, insulin remains the most effective therapy not only for type 1, but also for T2DM, and it is still impossible to substitute in a fraction of patients. Obviously, the availability of a wider spectrum of therapeutic agents, many of which are better tolerated than insulin, has reduced the field of application for insulin treatment; presently, insulin is used only in those who cannot maintain an adequate glycemic control with other drugs. The safety and tolerability of insulin treatment has been greatly improved over the past two decades, thanks to the identification of insulin preparation with more favorable kinetics, and to a better understanding of the most appropriate administration schemes. Furthermore, a lively research activity is currently ongoing, in order to make insulin therapy even safer and simpler for patients.
